# Achilles tendon compositional and structural properties are altered after unloading by botox

**DOI:** 10.1038/s41598-017-13107-7

**Published:** 2017-10-12

**Authors:** Hanifeh Khayyeri, Parmis Blomgran, Malin Hammerman, Mikael J. Turunen, Annika Löwgren, Manuel Guizar-Sicairos, Per Aspenberg, Hanna Isaksson

**Affiliations:** 1 Department of Biomedical Engineering, Lund University, Lund, Sweden; 2 Department of Clinical and Experimental Sciences, Linköping University, Linköping, Sweden; 3 Department of Applied Physics, University of Eastern Finland, Kuopio, Finland; 4 Swiss Light Source, Paul Scherrer Institut, Villigen PSI, Switzerland

## Abstract

Tendon function and homeostasis rely on external loading. This study investigates the biological mechanisms behind tendon biomechanical function and how the mechanical performance is affected by reduced daily loading. The Achilles tendons of 16 weeks old female Sprague Dawley rats (n = 40) were unloaded for 5 weeks by inducing muscle paralysis with botulinum toxin injections in the right gastrocnemius and soleus muscles. The contralateral side was used as control. After harvest, the tendons underwent biomechanical testing to assess viscoelasticity (n = 30 rats) and small angle X-ray scattering to determine the structural properties of the collagen fibrils (n = 10 rats). Fourier transform infrared spectroscopy and histological staining (n = 10 rats) were performed to investigate the collagen and proteoglycan content. The results show that the stiffness increased in unloaded tendons, together with an increased collagen content. Creep and axial alignment of the collagen fibers were reduced. Stress-relaxation increased whereas hysteresis was reduced in response to unloading with botox treatment. Our findings indicate that altered matrix deposition relies on mechanical loading to reorganize the newly formed tissue, without which the viscoelastic behavior is impaired. The results demonstrate that reduced daily loading deprives tendons of their viscoelastic properties, which could increase the risk of injury.

## Introduction

The Achilles tendon has a predominantly mechanical function by enabling energy-efficient repetitive locomotion, such as walking and running. The tendon attaches the gastrocnemius and soleus muscles to the calcaneal bone, and withstands large forces^1^. Aging and sport activities make the tendon prone to injuries and often complete ruptures^2–4^. However, management and rehabilitation schemes after ruptures are debated, as there is a lack of knowledge in how external loading affects tendon restoration.

The constituents that make up the tendon are mainly water (70%), collagens (collagen type I ~90% of dry weight) and proteoglycans (~2–5% of dry weight)^5–7^. The rest of the tendon consists of small amounts of elastin and other molecules. The collagen fibers and fibrils are organized as parallel strands along the loading axis, resulting in an anisotropic tissue matrix with high mechanical strength during axial tension^8^ and reduced strength under compression or shear^9^. The Achilles tendon is viscoelastic, which defines time-dependent functional characteristics that describe the recruitment of collagen fibers (creep), molecular interactions and fluid exudation (stress relaxation), and energy up-take (hysteresis). Tendon cells (tenoblasts and some mesenchymal stem cells^10^) reside between the strands of collagen fibers where they can sense physical stimuli, like fluid flow, and change their synthetic activities accordingly^11^. The response to this mechanosensation means that the tissue composition, structure and biomechanics adapts to the local mechanical environment^11,12^.

Mechanical loading can have both detrimental and positive effects on tendon homeostasis, since both increased and reduced magnitude of loading can lead to up-regulation of inflammatory signals that are similar to those in chronic tendon injuries^13–15^. Nonetheless, some loading magnitudes promote collagen production^16^ and reduce adhesion of the collagen fibers to each other. Thus, there is a window of mechanical loading that is beneficial for Achilles tendon health. In order to better understand this range of loading, we need to unravel the role of mechanical loading on Achilles tendon homeostasis; one way to achieve this is by investigating the effect of unloading of tendons.

Studies using models of unloading or disuse are sparse and have reported contradictory results. For example, *in vitro* studies on canine patellar, flexor and rabbit Achilles tendons have shown reduced mechanical integrity, collagen production and matrix degeneration due to immobilization^17–21^. In contrast, *in vivo* studies on Achilles tendons have discovered that the level of exercise does not change tendon stiffness^22,23^, whereas others have reported reduced stiffness due to immobilization *in vivo* ^24–26^. Additionally, it has been suggested that the change in stiffness depends on the degree of disuse and time, where the low stiffness found after a short period of immobilization may turn into an increased stiffness after a longer period of disuse^27^. Another approach is to study the effect of increased loading, where for example uphill running has been shown to increase tendon modulus in rats^28^. Considering the contrasting research findings, it is not surprising that clinical treatment of tendon injury is controversial. Thus, more comprehensive investigations are necessary to understand the basic synergies between tendon composition, structure and mechanical function, focusing on the role of viscoelastic properties.

In this study, we hypothesized that mechanical unloading impairs the viscoelastic behavior of the Achilles tendon, leading to reduced creep, hysteresis and increased stress-relaxation. Additionally, we hypothesized that unloading would lead to reduced collagen content and a more disorganized tissue matrix, which would reflect and explain the changes in the viscoelastic behavior. Mechanical tests were performed to measure the viscoelastic properties of the Achilles tendon. Fourier transform infrared spectroscopy and small angle X-ray scattering were used to investigate the tendon molecular composition and organization of the collagen fibrils. The study was complemented with histology of collagen and proteoglycans. The animal model was based on growing Sprague Dawley rats.

## Results

### Biomechanical characterization

**Figure 1:**
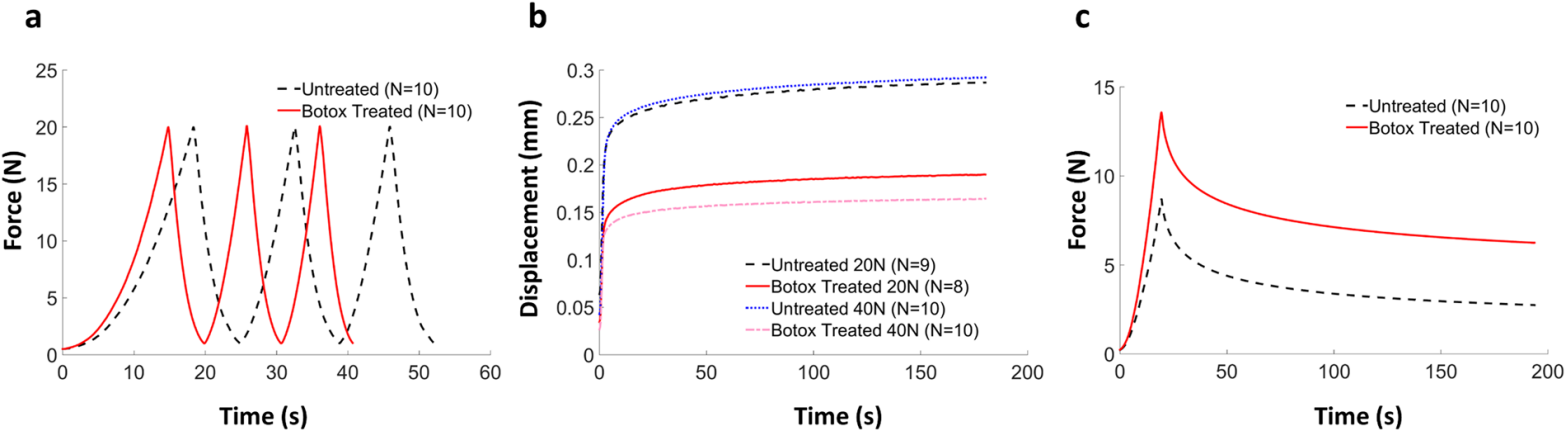
Experimentally observed behaviour of cyclic loading (3 cycles) **(a)** creep at 40 N (**b**) and stress-relaxation (**c**). The curves show averaged the data.

**Figure 2:**
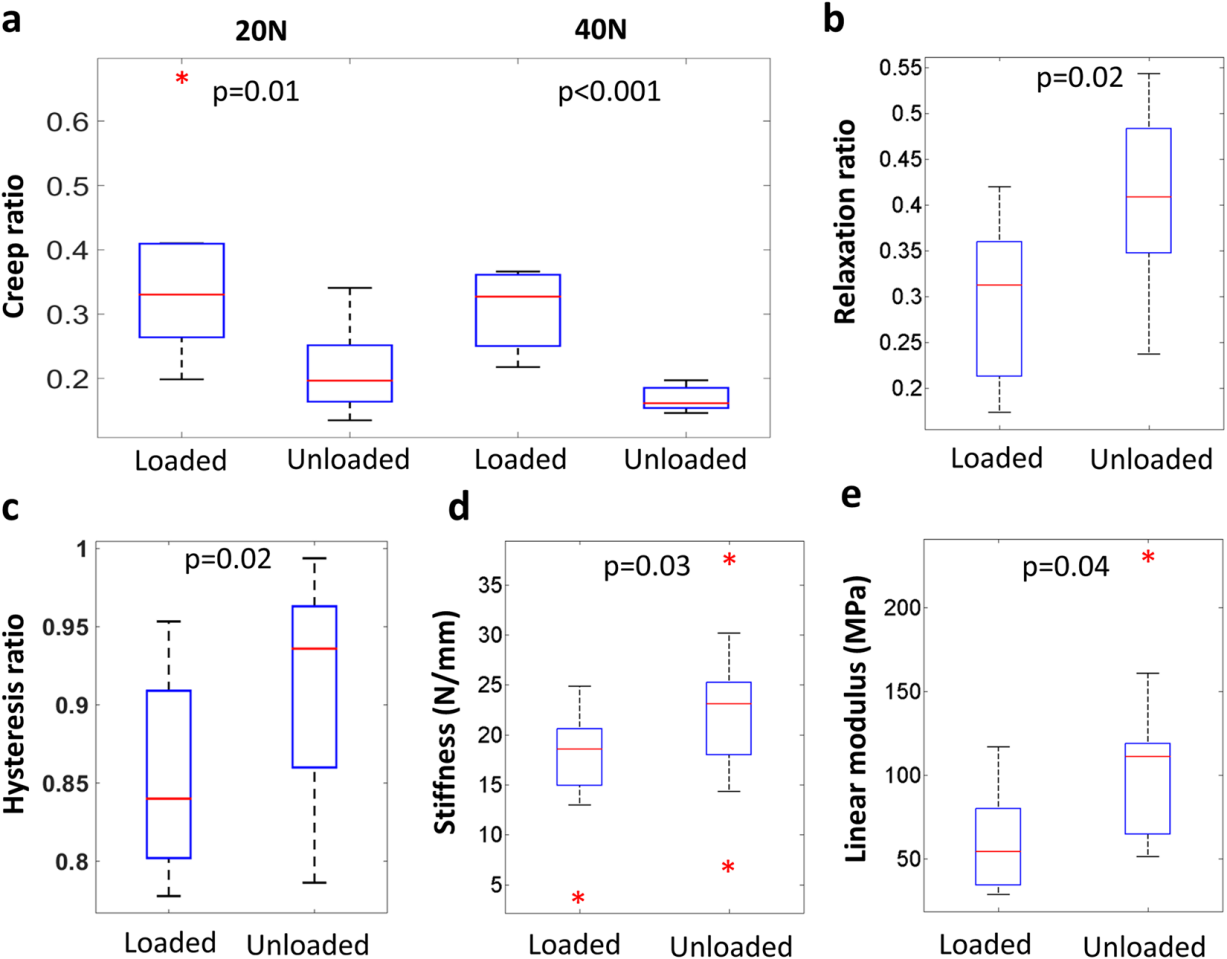
Unloading during 5 weeks lead to decreased creep ratio (**a**) but increased relaxation ratio (**b**). The energy-absorption capacity was lower in unloaded tendons as reflected by an increased hysteresis ratio (**c**). However, unloaded tendons exhibited a stiffer behavior (**d**,**e**). Wilcoxon signed rank (paired) test was used, and the boxplots mark the medians and the variation of the data (*are outliers) (n = 10 in all groups).

Different mechanical tests were conducted to retrieve information about the biomechanical behavior of tendons as a functional material. Viscoelastic creep, stress relaxation and hysteresis were quantified after 5 weeks of unloading (see Fig. 1 for average mechanical testing results). The results showed that the creep ratio (final elongation/original length) decreased in response to unloading. This was seen at both 20 N and 40 N of tension (Fig. 2a). The unloaded tendons exhibited an increased relaxation ratio (maximum longitudinal stresses/minimum longitudinal stresses; Fig. 2b). This means that when the tissues were stretched and held at a constant displacement, the force within the unloaded tendons decreased more than in the loaded tendons. Further, hysteresis was investigated in cyclic tensile loading of the tendons as it describes the damping capacity of the tissue. When reported as a ratio between loading and unloading curve removes any variability between the speciemens in measure peak force or displacement^29^, and using this an ideally linear elastic material would have a hysteresis ratio of 1.0. The results showed that the viscoelastic damping property was reduced following a period of unloading, as reflected by an increased hysteresis ratio (Fig. 2c). Thus, immobilization of Achilles tendons affected all three measures of viscoelastic properties.

The elastic properties of the tendons, measured from the linear region in the first cyclic load curve, were also affected by unloading. The tendons became stiffer and had a higher linear modulus as an effect of the treatment (Fig. 2d,e). There was no difference in the cross-sectional areas between the unloaded and the contralateral loaded tendons (loaded: 2,5 (0,5) mm^2^ and unloaded: 2,5 (0,7) mm^2^).

### Structural characterization

**Figure 3:**
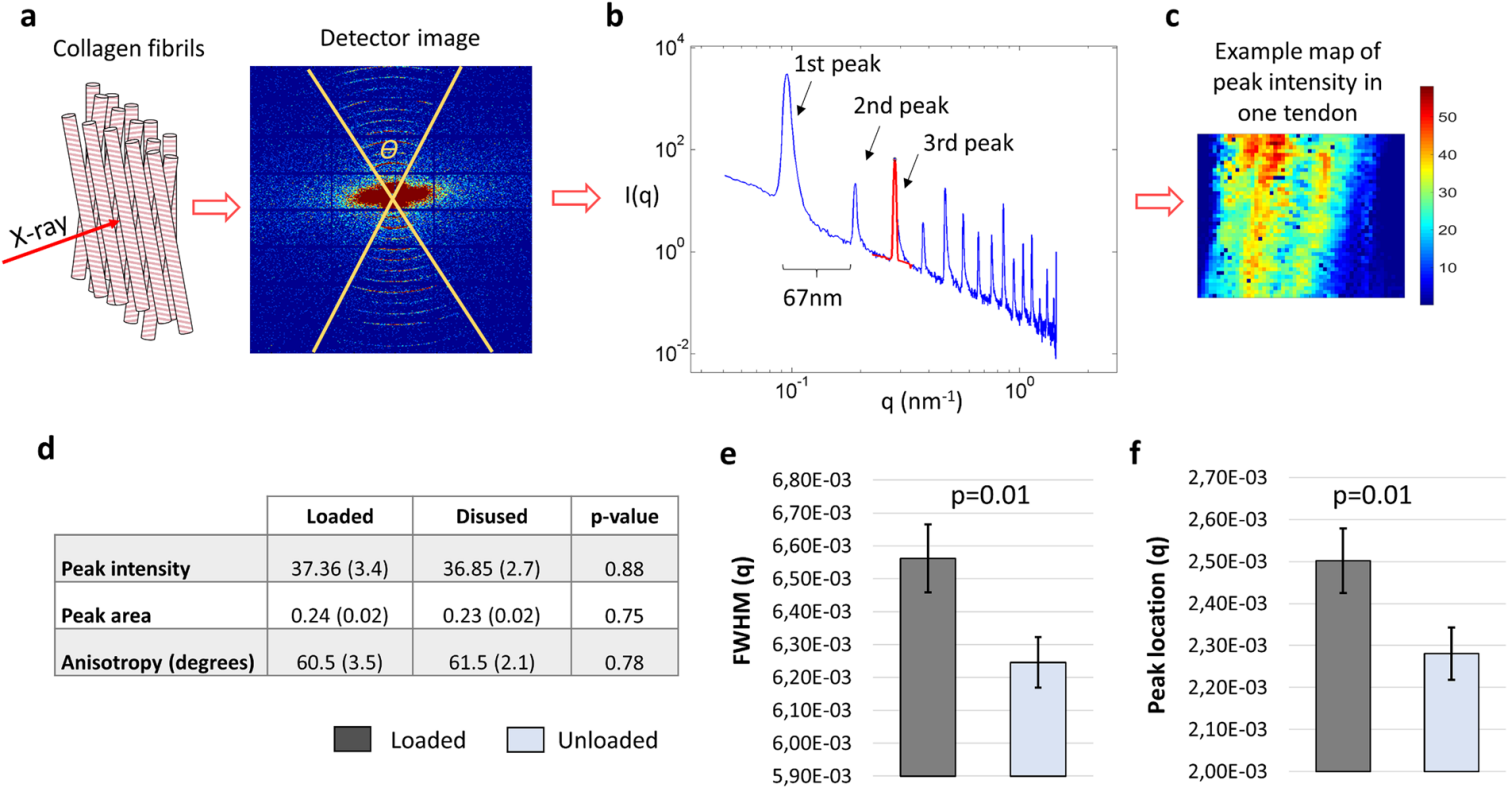
SAXS: A region in the middle of the intact tendons was radiated with X-rays. The resulting X-ray diffraction pattern (detector image) contains information on the collagen organization. The diffraction pattern of tendon collagen was typically concentric arcs from which the tissue structure was computed (**a**). Angular integration of each diffraction pattern, in the interval denoted *Ɵ*, generated an intensity curve versus *q* where the spikes are the collagen peaks arising from the D-periodicity of the molecule (**b**). By analyzing the 3^rd^ collagen peak for each data point from the measured region, spatial information about the tissue organization could be calculated (**c**, example of spatial map). It was found that unloading led to more fibrillar adhesion (**d**,**e**) Full Width Half Maximum - FWHM), but no major change in fibril organization. Furthermore, the peak location q (=2π/D-period) was slightly lower in unloaded tendons (**f**). This illustrates larger D-spacing compared to loaded tendons. A mixed effect model with likelihood ratio test was used to distinguish statistical difference between the loaded and unloaded tendons, where the bar plots display the estimated averages and standard errors (n = 10 in both groups).

The highly organized and periodic structure of the collagen fibrils (normally D-period of 67 nm in wet type I collagen) was visualized using small angle X-ray scattering (SAXS; Fig. 3a). By azimuthally integrating the scattering pattern on the detector image over *θ* (degrees), the intensity *I(q)* curve was obtained and a Gaussian curve was fitted to the 3^rd^ order collagen peak (Fig. 3b). Information about collagen organization (peak intensity and anisotropy), fibrillary adhesion (measured as full-width-at-half-maximum (FWHM), see^30^) and packing (peak location, D-spacing) were extracted from the curve fit. By measuring a defined region of the tendon, spatial maps of the structural parameters were created for each tendon (e.g. Figure 3c).

SAXS confirmed that both loaded and unloaded tendons were composed of collagen fibrils that were highly aligned (Fig. 3d. Peak intensity) with a similar degree of fibril dispersion (Fig. 3d. Anisotropy) regardless of load environment. Unloading led to reduced FWHM, indicating more fibrillar adhesion (Fig. 3e). The treatment also resulted in lower D-spacing compared to the loaded group (Fig. 3f). Shorter D-spacing is believed to be a result of shrinkage^31,32^ or fibrillar damage^33,34^. Goh *et al*.^31^, argue that D-spacing is positively correlated with fibril diameter, however Lavagnino *et al*.^35^ have shown that changes in diameter distribution are not reflected in the mechanical properties. Assuming that collagen periodicity affects mechanical properties, then the lower D-spacing in loaded tendons compared to unloaded would illustrate that the fibrils have a more coiled configuration in mobilized tendons.

### Molecular characterization

**Figure 4:**
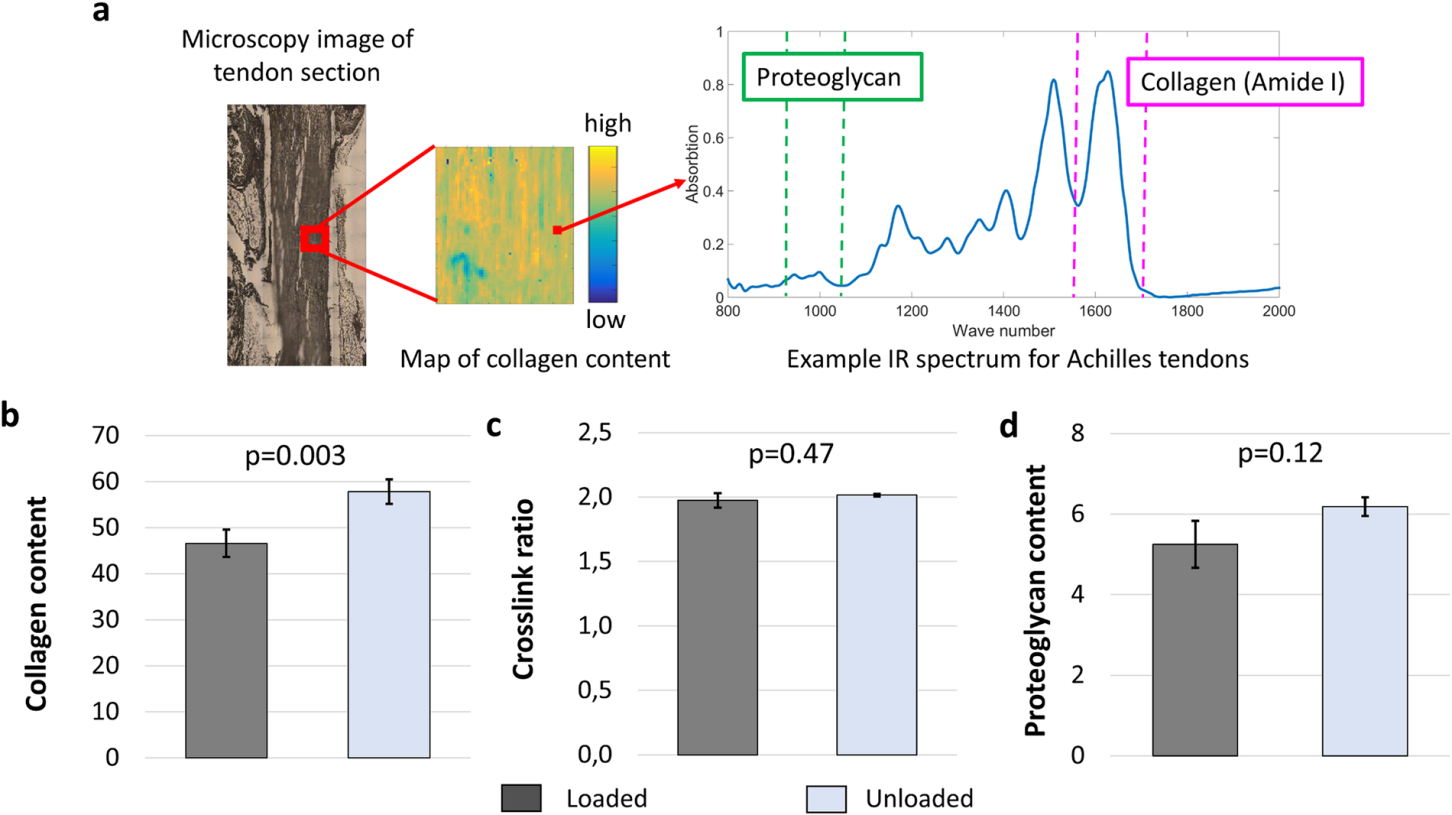
A region of the mid-tendon section is selected using a light microscope and measured with IR spectroscope that creates a spatial map of the molecular content in that region. Each of measurement point is associated with an absorption spectrum from which collagen and proteoglycan contents are estimated (**a**). Collagen content (absorption of peak 1720–1585 cm^−1^), estimated as the stretching vibrations of the C=O amide bond, increased in response to unloading (**b**). Unloading did not affect cross-link ratio (ratio of peaks at 1660/1690 cm^−1^) (**c**) and resulted in a slight but not significant increase in proteoglycan content (absorption of peak 1125–970 cm^−1^) (**d**). Absorption units are arbitrary. A mixed effect model in combination with a likelihood ratio test was used to distinguish statistical difference between the loaded and unloaded tendons, where the bar plots displays the estimated averages and standard errors (n = 10 in both groups).

Absorption spectra collected with Fourier transform infrared (FTIR) spectroscopy of thin sections of the tendons were used to map the spatial molecular composition. Specifically the Amide I peaks were analyzed, from which collagen content and cross-link ratio was estimated (Fig. 4a). This showed an overall higher deposition of tissue matrix in the unloaded tendons, since an increase in collagen content was measured (Fig. 4b). However, unloading did not affect collagen crosslink ratio (Fig. 4c), which attributes to collagen maturity. There was noticeably larger variability in cross-link ratio in loaded tendons that could be a sign of higher collagen turnover. Proteoglycan content, estimated at lower wavelengths (1125-970 cm^−1^)^36,37^, increased also but without statistical power (Fig. 4d).

### Histology

**Figure 5:**
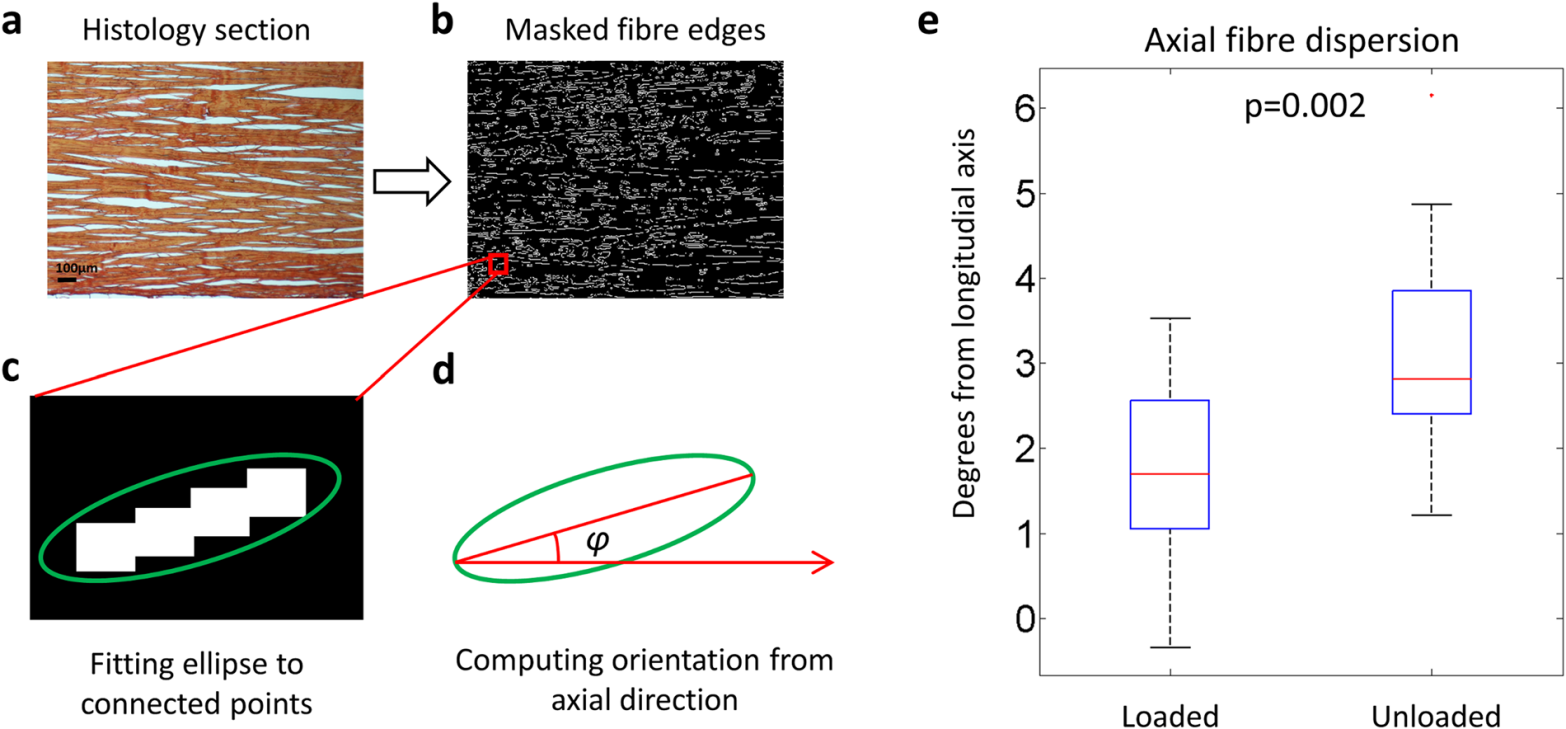
The custom-made script for histological analysis. The microscopy image (**a**) of the histological section was thresholded and filtered to identify fiber edges (**b**). An ellipse was fitted to the connecting points that consisted of more than 18 connected neighboring points (**c**) and the fiber orientation, *φ*, was measured as the angle from the axis of loading (**d**). Wilcoxon signed rank test (paired) showed significantly (p = 0.002) reduced axial fiber organization due to unloading (**e**), where medians and variability are shown in the boxplots (n = 10 in both groups).

Identifying chemical compounds with histological staining is still the gold standard for tissue characterization. The tissue was prepared following standard histological protocols, sectioned and stained with picrosirius red for collagens and alcian blue for proteoglycans. A qualitative analysis of the histological section could not identify any clear effect on the Achilles tendons due to unloading. However, the sections were also used to investigate the microscopic structural changes by using a custom-made image analysis script in Matlab (Fig. 5a–d). This technique showed that unloading of Achilles tendons led to more disorganized collagen on the fiber level (Fig. 5e).

## Discussion

The results from the mechanical tests showed that daily mechanical loading is crucial for maintaining the viscoelastic behavior of Achilles tendons. The hypothesis of this study was partly corroborated since unloading led to reduced creep, meaning reduced collagen fiber recruitment during tension. Also the energy-storing capacity of the tendons, hysteresis, was reduced, which could potentially be a risk factor for tendon injuries^38,39^. In contrast to the hypothesis, we did not expect unloading to result in increased collagen content.

Our results showed that unloading of healthy Achilles tendons does not degrade the tissue matrix. Since the rats in this study are still growing^22^, new tissue is deposited. The lower collagen content in loaded tendons could be due to higher collagen turnover (balance of degeneration and deposition) as a process of continuous adaptation^40^. Collagens are the constituents that provide tendon with its mechanical tensile strength. Thus, increased collagen deposition in the unloaded tendon group could explain also the higher stiffness that was measured in this study. Increased collagen content following reduced external loading has also previously been reported in patellar^41^ and Achilles tendons^22^.

It may seem counterintuitive that loaded Achilles tendons are softer than the unloaded ones (in their linear behaviour). However, we argue that the unloaded tendons have adapted to the new mechanical environment. The physical forces and functional demands in biology can stimulate adaptation in the form and shape of biological matter. A healthy tendon has a dynamic mechanical function as the tissue is continuously subjected to cyclic loading and absorbs energy effectively. This requires the tendon to be compliant and maintain its viscoelastic properties to maximize muscle efficiency^42,43^. An unloaded tendon on the other hand is exposed to a mechanical environment that is similar to ligaments that function to provide stability. This requires static strength, which is gained by increased collagen matrix deposition that provides stiffness. Also in humans, it has been shown that static training leads to increased tendon stiffness whereas dynamic training does not^44^. Another study in human tendons has also reported that healthy tendons are softer and more compliant compared to tendons with tendiopathy^45^.

The increased stiffness findings disagree with some literature where tendon unloading has resulted in softer tendons. At a closer look, all the contradicting experiments use hind limb suspension models^24–26^ which potentially causes a different change in loading pattern compared to the Botox model used in this study. Tail suspended rats can cause vigorous loading when the animals scratch themselves between long periods of inactivity, while the muscle paralysis of the Botox model permits normal daily activity with low to moderate loading of the tendon though passive motion. By also characterizing the composition of the tissue, this study further explained that the increased stiffness can be a result of the higher collagen content found in the unloaded tendons. It should be noted however, that hind limb suspension might lead to different mechanical, compositional and structural findings. The experimental set up, using both limbs of the animal, could potentially affect the contralateral control tendon as it is subjected to more body weight which could influence remodeling metabolic activities. Nevertheless, the results from this study and others, using both load reduction models, show that reduced exposure to mechanical loading deprive tendons of their viscoelastic properties^22,26,46,47^. This can increase the risk of injury once the tissue is re-loaded.

Research has shown that Botox treatment can affect neuronal pathways, which in turn control neuropeptide release that regulates inflammatory pain sensitization^48,49^. Studies on injured ligaments have shown increased mRNA levels for collagen type I and type II after denervation^50^. But the tendons used in this study were healthy and we used intramuscular injections, which strongly binds Botox to the muscles^51^. Moreover, retrograde axonal transport has been described as slow and does not penetrate the brain barrier; thus the local Botox treatment in the muscle is not likely to have a distant effect on the tendon. In contrast, it is expected to have a clear effect on the magnitude of mechanical loading. Nevertheless, specific mechanisms of Botox on healthy growing tendons require more research. It is worth mentioning that this is not a clinical study and the findings presented are not yet ready for clinical applications. The animal model and cyclic loading protocol were developed in a previous study (please see^22^), and was not investigated. Moreover, the resting angle was not measured which together with complete unloading could further reduce the tissue strains; however this is difficult to control in experiments. The timing (5 weeks) was chosen to allow ample time for tendon remodeling and adaptation. Female rats were used to balance the fact that the animals are still growing and females have a more limited longitudinal growth from 10 weeks onwards, compared to male rats. But the gender difference in response to loading is unfortunately not within the scope of this study. Following the 3 R principles for animal research, both limbs of the animals were used in this study. This could have resulted in animals compensating on the contralateral limb, which increases the magnitude of daily loading on the loaded tendons.

In this study, SAXS measurements were performed on the entire tendon rather than on a section of the tendon. This provides and average 2D spatial map of the collagen orientation in the chosen frontal plane, but does not capture any anteroposterior differences that could have existed in the tissue. A study on turkey tendon has however shown that the FWHM parameter can be used to measure collagen packing^30^, where decreased FWHM, as found in the unloaded tendons of this study, would show more densely packed fibrils. This also corresponds well with the findings of increased collagen production in the unloaded tendons. However, this variation is probably minor in intact tendons. FTIR does not measure absolute amounts of the specific molecular component such as collagen type I or elastin, which is possible with biochemical assays. Instead it measures the Amide I bindings. However, it could map out the molecular composition over a specific region. Although histological stains can bind to specific targets and the sections are easy to examine in a standard microscope, the staining technique is not sensitive to small changes and the results are believed to be affected by the sample fixation procedures. The code developed to analyze the fibre orientation from histology was developed to exclude as much artefacts as possible. Still, there is still a possibility that some artefacts are included in the analysis. However, any potential artefacts should effect both groups similarly. The histology samples were not tested mechanically before fixation and strain was also not controlled for (but was approximately zero; thus the correlation between histology and mechanical testing is unknown), when preparing the samples for histology. It should be also noted that all measurements in this study were randomized and blinded to avoid any bias.

Despite the above mentioned limitations, by characterizing the structure of the collagen using SAXS and histology, we found that unloading impaired the organization of the newly formed matrix along the loading direction. The collagen fibers were more unorganized in the unloaded tendons, which was reflected by the reduced creep in the mechanical tests and explains the less efficient recruitment process. The degree of anisotropy at the fibril level did not change following load reduction. This parameter was calculated locally for each scan spot and could not be related to a global axis orientation, in contrast to the histological analysis. Thus, it does not entail information about fibril organization in the load-direction but describes fibril alignment in respect to each other. Nonetheless, the SAXS data revealed that the fibrils were more delaminated in the loaded tendons. Delamination of collagen fibrils (longitudinal separation of collagen fibrils) is a process that occurs during mechanical loading and fibrils stay delaminated even after the load has been removed^32^. This separation prepares the tissue fiber to carry high stresses at high strain rates, for example during a launch or startle responses. At high strain rates there is little time for interfibrillar sliding and there are higher viscous forces acting on the fiber. Delamination of fibrils maximizes the load-bearing capacity of the collagen and reduces the risk of damage, as shown in a study on the collagen of skin^32^. Future *in situ* diffraction analysis could shed more light on the micro structural damage mechanisms^33,34,52^.

The unloaded tendons showed increased stress relaxation, which means that there is more interfibrillar sliding. This behavior can be ascribed to proteoglycans which are believed to be responsible for fibrillar sliding. These molecules also bind water, which leads to swelling that can result in molecular changes in the collagen structure and create tension in the fibrils^53^. The stress relaxation response of a biphasic material, such as the tendon, is believed to be partly due to fluid exudation^17,54^. Therefore, the increased relaxation observed in the unloaded tendons could be due to higher fluid content that is exuded during the mechanical tests. We did not measure fluid content in this study, which is a limitation. The tendon contains a small amount of interfibrillar molecules, such as elastin and proteoglycans, and cross-links that bind the collagens. Elucidating the synergies between these molecules, the water and the collagen fibrils remains a key challenge for the future, as the interfibrillar matrix is believed to play a significant role in tendon health.

In conclusion, this study explains mechanobiological effects on young rat Achilles tendon function with remodeling of the tissue structure and composition. Our results showed that the structural organization of the Achilles tendon is a principal mechanism behind the dynamic function of the tendon. We also found that unloading with botox impedes tissue organization and therefore changes the viscoelastic response of the tendon. Additionally, we showed that the compositional changes have a direct effect on the static functional properties (e.g. stiffness) but play an indirect role on tendon dynamics (e.g. damping). Altered matrix deposition relies on mechanical loading to reorganize the newly formed tissue, without which the viscoelastic behavior is impaired. These findings suggest that the viscoelastic mechanical characteristics of the Achilles tendon could potentially be used to assess tendon health.

## Methods

### *In vivo* experiment

Female Sprague Dawley rats (n = 40 rats) aged 16 weeks (mean ± SD: 328 ± 19 grams) were used in this study. The right calf muscle (gastrocnemius muscle) was injected with 1U Botulinum Toxin type A (Botox) in three different sites to induce muscle paralysis and restrict the rats from loading their Achilles tendon. The left leg was left untreated and acted as control (loaded tendons). The rats were allowed to move freely in their cages for 5 weeks before their Achilles tendons were harvested. The harvested tendons were characterized with mechanical testing (n = 30 rats) and FTIR and SAXS experiments for tissue composition and organization respectively (n = 10 rats).

The tendons for mechanical characterization were harvested with the calcaneal bone and the gastrocnemius muscle. The Achilles tendons for compositional and structural evaluation were carefully dissected from the distal end of the calcaneal attachment together with the gastrocnemius muscle and plantaris tendon. The muscle was then scraped off and the plantaris was removed before the Achilles tendons were needled onto silicon gels and stored in formaldehyde awaiting further evaluation.

### Mechanical testing

The tendons were harvested with the calcaneal bone and the gastrocnemius muscle (average weight at harvest was: 328 ± 19 g (n = 40)). The muscle was carefully scraped off before it was clamped to the mechanical testing machine with sand paper. The tissue was fixed between the clamps with an angle between the tendon and the heel bone that corresponds to 30 degrees dorsiflexion. The tendon lengths and diameters were measured with a slide caliper before the tests were started. Sample-specific lengths were used for all measurements. The tendons were preloaded with 0.1 N before the start of each sample-test. Three different mechanical set-ups were performed in a material testing machine (100 R, DDL, Eden Pairie, MN), using a 445 N-capacity load cell. The data acquisition rate was 0.03 s.

1. *Cyclic loading (n* = *10 rats):* The tendons were loaded between 1–20 N for 20 cycles with a rate of 0.1mm/s. After the cyclic loading, the tendons were pulled to failure.
2. *Creep (n* = *10 rats):* The tendons were pulled to 20 N (approx. 25% of failure load) with a rate of 1mm/s kept at constant load for 300 s. After this, the tendons were rested for 3 hours before another creep test was performed. During the second test, the tendons were loaded to 40 N (approx. 50% of failure load), 1mm/s, and kept at the constant force for 300 s.
3. *Stress relaxation* (*n* = *10 rats*)*:* The tendons were pulled to 2mm displacement (approx. 20 N and 25% of failure load) with a rate of 0.1mm/s and kept at the constant displacement for 200 s.

The samples were kept hydrated by dripping saline on the tendons during the mechanical tests. All tendons were kept horizontally in gauze drenched in saline and covered with plastic to avoid evaporation immediately after harvest. Different biomechanical functional properties were calculated from the tests. These were:

- Cross-sectional area: the medial-lateral tendon diameter was used to compute the cross-sectional area of the tendon. The tendon was assumed to be cylindrical.
- Stiffness: measured from the slope of the first loading curve (force-displacement curve) in the cyclic test. This was also used to compute the linear modulus together with the cross-sectional area.
- Hysteresis: measured from the cyclic test. It was calculated as the area between the load and unload curve in each cycle, and is reported as the ratio between the loading and unloading area. Since the behavior of the first load cycle is affected by tendon crimp (wavy pattern of the collagen fibers that are straightened during tension), cycle 2–20 were used.
- Creep ratio: the ratio between the displacement after 300 s of load and the original tendon length (same length measurement was used for creep at 20 N and 40 N since no tissue elongation was measured between the tests). Two specimens in the botox-treated group and one in the loaded group were excluded from the analysis of the 20 N creep test, due to slippage in the grips.
- Relaxation ratio: the ratio between the force when 2mm displacement was reached and the force after 200 s of loading.

All tendons broke at the clamp during the failure tests and thus the measurements did not represent tendon peak force and were therefore excluded from the analysis.

### Small angle X-ray scattering

The tendons (n = 10 rats, stored in formaldehyde) were measured in whole with synchrotron light at the coherent small angle X-ray scattering (cSAXS) beamline at the Swiss Light Source (SLS), Paul Scherrer Institut (PSI), Villigen Switzerland^55,56^. Small pockets were made out of Kapton film. To keep the tendons moist, the samples were placed inside the Kapton pockets filled with formaldehyde before they were attached to the sample holder. The tendons had a diameter of about 1.7 mm.

Tendons were raster-scanned with 30 × 30 µm^2^ spot size and with scanning steps of 30 × 30 µm^2^ in a continuous line-scan mode. A Pilatus 2 M detector^57^ was used to record the 2D scattering patterns. An exposure time of 50 ms and X-ray wavelength of 1.0 Å were used. The sample-detector distance (7.11 m) and the beam center on the detector were calibrated using a Silver-Behenate (AgBH) powder standard. For each sample, a background measurement was performed on the Kapton pocket of the sample. The scattering due to Kapton in the analyzed q-region was negligible. The data was background corrected by subtracting the background measurements, which were taken in regions adjacent to the sample in order to include the effect of both Kapton and formaldehyde. Sample measurement areas were selected using a camera with a calibrated distance to the X-ray beam. Pixels blocked by the beam stop, dead or hot pixels, and insensitive gaps between the detector modules were excluded from all analysis using an image mask. The number of pixels analyzed were on average 5880 (±1812, SD) per tendon sample.

The *I(q)* scattering curves (e.g. Fig. 3b), containing information about the collagen structure and orientation, were obtained by azimuthally integrating the detector images over *θ*, where *θ* is the angle where the collagen rings were observed and it was calculated for each detector image from the angular full width of tenth maximum. For azimuthal integration, a mask for each detector image was created in the q-range of 0.05–1.45 nm^−1^ and the angular range *θ* in both collagen peaks separated azimuthally by 180 degrees. In the *I(q)-*curve, a Gaussian curve was fitted to the 3^rd^ order collagen peak in order (Fig. 3b) to determine collagen organization, alignment of the collagen fibers, and delamination of the collagen fibers. Anisotropy described fibril dispersion and was represented by angle *θ*. Peak location was measured as the radii of the 3^rd^ concentric arc in the detector image (Fig. 3a). D-spacing (periodicity) was then inversely proportional to peak location. The principal fibril direction was estimated with peak intensity where higher peak intensity represents more alignment. Peak full width of half maximum (FWHM) in the q-direction was used as measure of delamination of the collagen fibrils (see^30,32^ for proof of concept).

### Fourier transform infrared spectroscopy

For tissue composition (n = 10 rats), the tendons that had been measured with SAXS were further embedded in paraffin and 2 independent 3µm-thick sections from the middle cross-section of each tendons were placed on BaF_2_ windows and evaluated with the D7 FTIR spectroscopy beamline at Max-IV laboratories, Lund, Sweden.

A Bruker 66 V FTIR spectrometer coupled to a Bruker Hyperion 3000 IR microscope was used with a focal plane array detector. Based on the light microscope image, a region (340 × 340 µm, divided into 64 × 64 elements) representing the centre of the tendon was chosen for analysis using 64 scans and a spectral resolution of 8 cm^−1^. The infrared spectra were collected at the range of 4000 to 900 cm^−1^. Collagen content was estimated from the peak area under the Amide I peak (Amide I peak; 1720–1585 cm^−1^). The ratio between mature and immature collagen cross-links were estimated from the 1660/1690 cm^−1^ intensity ratio^58^. The proteoglycan content was estimated as the area under the peak located between 1125-970 cm^−1^ ^36,37^ (see Fig. 4a). Please note that the content measures are not absolute measures but relative measures. The calculated area that is estimating molecular vibrations (see Fig. 4a Example spectrum).

### Histological staining and analysis

The harvested tendons in formaldehyde were embedded in paraffin following standard a histological protocol with ethanol dehydration and histological clearing with HistoClear. The embedded tendons were sliced in 3 µm thick sections and the sections from the mid-tendon were selected for staining. Two sections were prepared for each staining and tendon. The sections were stained with Picrosirius red for collagen and Alcain blue (pH 2.5) for glycosaminoglycan (GAG). All sections were imaged and examined using a light microscope (Olympus BX50). A line profile was created for each image and the RGB profiles were quantified based on the intensity of the stains.

To extract fiber-orientation from the microscopy samples, the images were processed using the built-in functions in the Image processing toolbox in Matlab (R2014b, The Mathswork Inc., Natick, MA, 2000). The histological images were thresholded and the Canny edge detection was applied to find the collagen fibers. All connecting edges longer than 18 pixels were fitted to an ellipse respectively. The axes and the rotation of the ellipses were used to determine the axial fiber orientation (see Fig. 5a–d).

### Statistical analysis

Data normality was assessed in all cases. When data was not normally distributed, non-parametric test was used. Wilcoxon signed rank test (Matlab R2014b, The Mathworks Inc., Natick, MA, 2000) was used to analyze the biomechanical test results and the quantitative histology profiles. Since the FTIR and SAXS techniques generated many thousands data points per sample, a random slope model (mixed effect model) was used (R software, R Core Team, www.R-project.org, lme4-package). This model is only valid for normally distributed data, and all FTIR and SAXS data was normally distributed. Unloading was defined as the fixed effect and both treatment and rat were considered to have random effects on the outcomes (full model), *i*.*e*. the model considered intra- and inter-animal variability in response to loading as well as general biological variation. A likelihood ratio test was performed to calculate the p-values (χ^2^), where the null model was a model without the fixed effect (*i*.*e*. hypothesized that unloading has only a random effect like general biological variation). A probability level of < 0.05 was considered significant for all statistical tests. If the difference between the models (full model vs null model) was statistically significant, then that our fixed effect (unloading) was considered to have a statistically significant impact. Please observe that tissue harvest and tissue testing and characterization was carried out by different persons.

### Ethics statement

All experiments were performed in accordance with relevant guidelines and regulations. The *in vivo* experiments were approved by local ethics authorities (Djurförsöksnämnden in Linköping, Dnr. 60–12).

### Code availability

The custom built computer scripts that were used to analyze the SAXS, the FTIR and the histology are still under development and documentation. Thus, they are not currently publicly accessible. Details on the code used in this paper are available from the authors on request and for earlier versions of the code used for characterization of mineralized tissue, the readers are referred to previous publications by the authors (see^55,58,59^).
